# An Update on Narrowband Ultraviolet B Therapy for the Treatment of Skin Diseases

**DOI:** 10.7759/cureus.19182

**Published:** 2021-11-01

**Authors:** Elisha Myers, Shiva Kheradmand, Richard Miller

**Affiliations:** 1 Medicine, Florida Atlantic University Charles E. Schmidt College of Medicine, Boca Raton, USA; 2 Dermatology, Hospital Corporation of America/University of South Florida Morsani College of Medicine: Largo Medical Center, Largo, USA

**Keywords:** narrowband uv-b, covid-19, mycosis fungoides, atopic dermatitis, psoriasis, vitiligo, nb-uvb, phototherapy

## Abstract

The objective of this review is to provide an update on narrowband ultraviolet B (NB-UVB) as a treatment for various skin conditions. NB-UVB works by suppressing the cutaneous cell-mediated immune response and has been shown to be an efficacious and clinically tolerable treatment for a range of inflammatory dermatoses. A literature search was conducted by advanced searches of PubMed for NB-UVB treatment of dermatologic skin diseases with a focus on reports from 2010 to 2021, including both office-based and home-based phototherapy (HBPT). Data were prioritized based on studies with a high level of evidence using the Oxford Evidence-Based Medicine guidance.

We found that NB-UVB continues to serve as an effective form of therapy for several cutaneous conditions, including vitiligo, psoriasis, atopic dermatitis, mycosis fungoides, and other inflammatory dermatoses. The recent introduction of Janus kinase inhibitors in combination with NB-UVB suggests future promise in the treatment of vitiligo. Despite its rise in popularity, a decline was seen in office-based NB-UVB treatment during the coronavirus disease 2019 pandemic. Options are available to deliver NB-UVB at home with comparable efficacy to office-based treatments. In conclusion, for a select group of patients and conditions, NB-UVB continues to serve as an effective treatment modality with minimal side effects, with HBPT serving as an option to improve patient compliance.

## Introduction and background

The use of ultraviolet (UV) light as a treatment for skin disease was initially studied in 1903 by Dr. Niels Finsen, and later in 1925 by Goeckerman. UV phototherapy uses a selective range of UV light, which can be categorized into ultraviolet A (UVA), psoralen ultraviolet A (PUVA), and ultraviolet B (UVB) irradiation ranges. The UVB category of phototherapy includes broadband UVB (BB-UVB) at 280-320 nm and narrowband UVB (NB-UVB) at 311-313 nm. In comparison to previously mentioned UV phototherapy, NB-UVB has been shown to be more clinically tolerable with fewer side effects [1,2]. The exact pathophysiology of NB-UVB is multifactorial and complex [3]. In the treatment of inflammatory dermatoses, NB-UVB leads to a decrease in pro-inflammatory cytokines, a decrease in antigen presentation through inhibition of Langerhans cell activity, and a cascade of biological events which result in suppression of the cutaneous T-cell-mediated immune system.

Over the past several years, NB-UVB has proven to be an effective treatment option for conditions such as vitiligo, psoriasis, mycosis fungoides, atopic dermatitis, pruritus, photodermatoses, and other skin conditions. It can be used as monotherapy or in combination with topical or systemic agents. Despite its rise in popularity, in the era of the coronavirus disease 2019 pandemic, there was an initial decline in office-based NB-UVB treatments due to concerns of increased viral exposure [4]. Recent reports demonstrate that home-based phototherapy (HBPT) can serve as an option for a select group of patients, with efficacy comparable to office-based phototherapy. HBPT can be delivered in the form of booths and hand or foot unit devices with timers to prevent overexposure to UV light. HBPT has been shown to increase treatment compliance and may serve as a more cost-effective utilization of healthcare resources [5,6].

## Review

Narrowband ultraviolet B monotherapy for vitiligo

NB-UVB is considered a first-line treatment for patients with vitiligo. Areas affecting the face, neck, trunk, and proximal extremities are generally more responsive to NB-UVB compared to the distal extremities. Although the mechanism behind repigmentation with NB-UVB is not fully understood, it is believed to be caused by a decrease in inflammatory cytokines followed by a stimulation of melanocytes within the hair follicle [7]. In the treatment of vitiligo, NB-UVB has no significant difference in efficacy compared to PUVA or 308 nm-excimer laser [8]. A recent retrospective review of 58 patients who received NB-UVB therapy showed persistent skin repigmentation in 80% of patients one year after treatment in those predominantly suffering from non-segmental vitiligo [9].

Narrowband ultraviolet B combination therapy for vitiligo

A retrospective cohort of 109 patients compared twice-weekly NB-UVB therapy to a more intensified thrice-weekly treatment of NB-UVB therapy along with topical fluticasone propionate and/or tacrolimus [10]. Results showed both treatment groups demonstrated similar results, with a faster onset of repigmentation seen in the combination group. Recently, a randomized controlled trial of 517 patients found that combination therapy with NB-UVB therapy (hand-held unit) and mometasone was more effective than corticosteroid use alone. Response rates were seen in one-fourth of patients with better response to vitiliginous lesions on the head and neck [11]. Further, three recent systemic reviews showed topical tacrolimus and NB-UVB combination to be superior to NB-UVB alone [12-14]. Another meta-analysis demonstrated no significant difference between NB-UVB monotherapy versus combination NB-UVB with calcineurin inhibitors [15].

In a recent study, treatment with Janus kinase (JAK) inhibitors in combination with NB-UVB for vitiligo showed promising results. A retrospective case series of 10 patients found that low-dose NB-UVB (office-based and HBPT) or sun exposure was required in conjunction with oral tofacitinib to achieve repigmentation. The five patients who achieved repigmentation with oral tofacitinib all had exposure to either low-dose NB-UVB or sunlight [16]. Another case series of two patients showed greater than 75% repigmentation of the face with oral tofacitinib and NB-UVB [15]. A recent case report of a four-year-old boy showed complete repigmentation of segmental vitiligo using tofacitinib cream and handheld NB-UVB. Similarly, a pilot study of 11 vitiligo patients demonstrated a mean improvement of 70% in facial vitiligo after treatment with NB-UVB in combination with tofacitinib cream [17,18]. The Vitiligo Working Group’s recent recommendations were summarized by Kim et al. and can be helpful for clinicians to refer to as a guide for frequency, dosing, safety monitoring, and tapering of NB-UVB [17].

Home-based phototherapy for vitiligo

The use of HBPT for vitiligo is considered experimental by most insurance companies, yet it has shown efficacy in several clinical trials. A pilot randomized controlled study of 100 patients found HBPT to be as effective as in-patient phototherapy [19]. A significant cost reduction was also seen for patients. Importantly, adverse effects seen in treatment were more common with HBPT, indicating a need for further education in patients using HBPT. A similar cross-sectional study with a smaller sample of 18 vitiligo patients found similar results, with comparable levels of pigmentation improvement seen in HBPT compared to in-patient phototherapy [20]. Both studies noted a slight increase in side effects and the more cost-effective nature of HBPT.

Narrowband ultraviolet B therapy for psoriasis

For the treatment of psoriasis, NB-UV decreases the production of inflammatory cytokines and T-cells in the Th1 pathway and reduces keratinocyte proliferation [21]. NB-UVB-induced increase in serum vitamin D levels in patients with low serum vitamin D with psoriasis or AD has also been studied. The correlation between serum vitamin D levels to the severity of psoriasis and AD remains controversial [22-24]. NB-UVB is considered a first-line treatment for stable, moderate-to-severe plaque psoriasis affecting greater than 10% of body surface area in children and adults, with efficacy comparable to PUVA phototherapy. PUVA has been shown to be more effective for the treatment of palmoplantar pustular psoriasis and refractory plaque psoriasis, and xenon monochloride (XeCl) excimer laser is utilized for recalcitrant localized psoriasis affecting less than 10% of the body surface area [25,26]. For palmoplantar psoriasis, a study of 66 patients comparing the use of NB-UVB to UVA1 found that both treatment methods led to a statistically significant reduction in psoriatic lesions. In the same study, 68.8% of patients in the UVA1 treatment group showed marked improvement in comparison to 34.4% in the NB-UVB phototherapy group [27]. Randomized controlled trials have shown combination therapy of NB-UVB with methotrexate leads to higher percentage clearance with a lower recurrence rate when compared to methotrexate alone [28].

Narrowband ultraviolet B therapy for mycosis fungoides

NB-UVB is considered first-line therapy for early-stage mycosis fungoides, with no significant difference compared to PUVA phototherapy in the treatment of early-stage disease [29-32]. Studies have demonstrated variable median relapse-free intervals with NB-UVB [33]. Moreover, recent data suggest that NB-UVB has the potential to serve as a disease-modifying therapy with long-term disease-free survival. A cohort study of 117 patients with stage I mycosis fungoides treated with a single course of NB-UVB showed an 80% complete response rate and a 60% disease-free survival of more than five years after treatment. Patients more likely to achieve this outcome were under the age of 50 and had stage IA disease [34].

Narrowband ultraviolet B therapy for atopic dermatitis

The use of phototherapy in AD works by the suppression of Th2 immune response, improvement of the skin barrier, and decrease in skin infections [35]. Current guidelines for the treatment of AD recommend NB-UVB as second-line therapy for children and adults with acute or chronic AD not responsive to topical corticosteroids or immunomodulators. Three retrospective studies showed a greater than 60% reduction in the severity of AD in children [36-38]. Further, in one review, clearance of AD was maintained among more than 50% of patients after 12 months [38], in contrast to 66% who relapsed after three months in another review [37]. Systematic reviews continue to support NB-UVB as an effective form of phototherapy in the treatment of AD when compared to other light-based treatments [39,40].

Narrowband ultraviolet B therapy for pityriasis lichenoides et varioliformis acuta and pityriasis lichenoides chronica

NB-UVB is a treatment modality commonly used for pityriasis lichenoides et varioliformis acuta (PLEVA) [41], a relatively uncommon chronic inflammatory papular disease, which is more severe than pityriasis lichenoides chronica (PLC). A systematic review regarding the use of phototherapy in the treatment of pediatric patients with PLEVA showed 73% lesion clearance with NB-UVB and no recurrence. In the same study, PUVA and BB-UVB showed a higher percentage of lesion clearance; however, both groups showed higher rates of recurrence [42]. Recently, a study of 29 patients with a diagnosis of PLC found complete response in 24 patients with NB-UVB treatment without relapse [43].

Narrowband ultraviolet B therapy for granuloma annulare

Generalized granuloma annulare (GA) is more responsive to NB-UVB compared to other subtypes of GA. Furthermore, it is well tolerated in patients suffering from this condition. Two retrospective studies of patients with generalized GA showed at least 50% clinical improvement in more than half of the patients when treated with NB-UVB [44].

Narrowband ultraviolet B therapy for pruritus

The cause of pruritus varies considerably. One way NB-UVB reduces the symptoms of pruritus is by decreasing the inflammatory cytokine, IL-31, involved in the itch pathway. Recent reviews support the use of NB-UVB in reducing pruritus for patients with uremic pruritus secondary to chronic kidney disease, cholestasis-induced pruritus, prurigo nodularis, HIV-induced pruritus, and other inflammatory dermatoses that cause chronic pruritus [45].

Narrowband ultraviolet B therapy for photodermatoses

Patients affected by photodermatoses such as solar urticaria and polymorphous light eruption (PMLE) can successfully respond to NB-UVB as a form of photoadaptation, demonstrating relapse in eruptive episodes. When treating these conditions, worsening of photodermatoses can occur which can be managed by topical corticosteroid and dose increment reduction [46]. A recent study of 39 patients with solar urticaria supported NB-UVB as an effective treatment for this condition, with patients experiencing a decline in recurrent episodes [47]. Similarly, two recent studies showed a significant reduction of PMLE episodes in patients undergoing NB-UVB therapy [48].

Narrowband ultraviolet B therapy for lichen planus

Phototherapy can be considered a treatment modality for patients with generalized lichen planus (LP). A randomized clinical trial of 46 patients comparing systemic corticosteroids to NB-UVB therapy found NB-UVB to be a significantly more effective treatment for generalized LP [49]. Another study involving 24 patients with generalized LP had two-thirds of their patients achieve partial or complete remission with NB-UVB therapy, while 33% did not respond to treatment [50].

Narrowband ultraviolet B therapy for pityriasis rubra pilaris

For NB-UVB treatment of recalcitrant pityriasis rubra pilaris (PRP), patients should undergo photoprovocation prior to therapy as there have been reports of photoaggravated PRP [51]. Data are limited regarding NB-UVB treatment of PRP and mainly in the form of case reports. A case report showed that a combination of acitretin in NB-UVB with oral acitretin led to near-clearance of PRP [52]. Another case report showed complete resolution after four months of NB-UVB for a case of PRP recalcitrant to oral isotretinoin [49]. Recently, another case report demonstrated 90% improvement after 19 sessions of NB-UVB in an eight-year-old with a generalized eruption of PRP [53,54].

Narrowband ultraviolet B therapy for acne

Very limited data exist for the treatment of NB-UVB therapy for acne. It has been suggested that UVB directly inhibits *Cutibacterium acnes* and affects the products of inflammatory cytokines. A case report by Zeichner et al. showed improvement in inflammatory papules and pustules in a pregnant patient after two months of NB-UVB therapy [55]. A recent study of 104 patients demonstrated that oral azithromycin in combination with NB-UVB led to improvement of inflammatory papules when compared to oral azithromycin alone [56].

Limitations

Limitations of this review include the wide breadth of information covered; therefore, not all reviews which supported information included in this paper were analyzed in this study. In addition, the review was limited to articles written or translated to English. Variability in treatment outcomes with NB-UVB can result from variation in dose delivery, the number of treatments, and subjectivity in minimal erythema dose testing in correlation with appropriate Fitzpatrick skin types.

## Conclusions

This review serves as a reminder to clinicians that NB-UVB is generally well-tolerated with few side effects and remains an effective treatment for different skin conditions in pediatric, adult, pregnant, and geriatric patient populations. NB-UVB continues to be efficacious in treating patients with vitiligo, psoriasis, mycosis fungoides, AD, photodermatoses, pruritus, generalized LP, generalized GA, PLEVA, and PLC. Recent studies show future promise in combination NB-UVB with topical JAK inhibitors for patients with vitiligo. In comparison to PUVA, NB-UVB has not been shown to induce carcinogenesis.

While office-based phototherapy serves as the main treatment for delivery of NB-UVB, home-based treatments can be considered as an efficacious option based on the evaluation of each patient’s unique situation. Numerous large studies have shown support for HBPT in the treatment of vitiligo. However, this option can be considered for all photoresponsive skin conditions. HBPT is not without its challenges which include patient training and education and insurance barriers. Additionally, future long-term studies in the form of large randomized controlled trials would solve any concerns about the safety of HBPT.
